# Conduction abnormalities after TAVI and their role in patient management: a systematic review

**DOI:** 10.3389/fcvm.2026.1706176

**Published:** 2026-02-13

**Authors:** Ionuț Tudorancea, Irene Paula Popa, Mihai Ștefan Cristian Haba, Ștefan Popa, Viviana Onofrei, Dragomir Nicolae Șerban, Ionela Lăcrămioara Șerban, Irina Iuliana Costache-Enache, Radu Iliescu, Cătălin Loghin

**Affiliations:** 1 Grigore T. Popa University of Medicine and Pharmacy, Iaşi, Romania; 2 Cardiology Clinic, “Saint Spiridon” County Clinical Emergency Hospital, Iaşi, Romania; 3 Transcend Research Centre, Iași, Romania; 4 Division of Cardiology, Department of Internal Medicine, UTHealth McGovern Medical School, Houston, TX, United States

**Keywords:** conduction abnormalities, ECG, pacemaker, PPM-I, TAVI

## Abstract

**Objective:**

Transcatheter aortic valve implantation (TAVI) is associated with a relatively high incidence of permanent pacemaker implantation (PPM-I). We aimed to evaluate the existing literature on electrocardiographic (ECG) changes before and after TAVI, identify predictors for PPM-I, and suggest a standardized post-TAVI ECG monitoring protocol.

**Methods:**

A systematic literature review was conducted across multiple databases, including PubMed, Web of Science, and JSTOR, to identify studies published between 2001 and 2024.

**Results:**

From an initial pool of 24,170 records, 17 studies met the inclusion criteria. Pre-existing right bundle branch block and significant prolongation of ECG intervals were identified as strong predictors of PPM-I. Following TAVI, new-onset left bundle branch block, prolonged PR interval, and QRS complex widening were the most common ECG changes.

**Conclusion:**

Systematic periprocedural ECG monitoring during TAVI is of paramount importance for the early recognition of conduction abnormalities (CAs) that predict the need for PPM-I.

## Graphical Abstract

**Figure:**
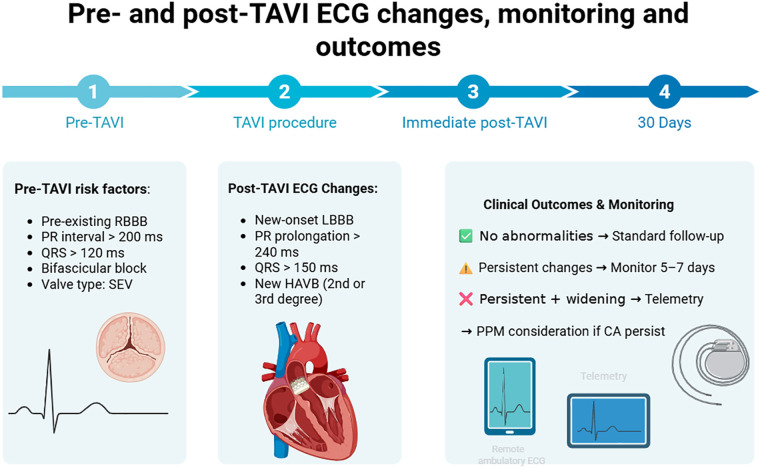
Created in “Biorender” (https://www.biorender.com/).

## 1 Background

Aortic stenosis (AS) is one of the leading causes of cardiovascular morbidity and mortality in individuals over 60 years of age, with its prevalence expected to increase (1). Transcatheter aortic valve implantation (TAVI) is currently the standard of care for patients with severe AS who are at high or prohibitive surgical risk (2, 3). Current randomized studies have expanded the indications for TAVI, showing its effectiveness in patients at both intermediate and low surgical risk (4, 5). Despite its benefits (i.e., higher survival rates, lower morbidity, and improved outcomes), TAVI is associated with a significant incidence of ECG conduction abnormalities, such as bundle branch blocks and atrioventricular block, which may necessitate permanent pacemaker implantation (PPM-I) (6, 7). The anatomical configuration of the cardiac conduction system makes it particularly susceptible to mechanical interaction during TAVI. The His bundle and proximal left bundle branch course immediately beneath the non-coronary and right coronary cusps, traversing the thin membranous septum adjacent to the aortic annulus—the precise region where both balloon-expandable and self-expandable prostheses exert radial force. Therefore, factors such as valve deployment, annular or left ventricular outflow tract calcification, and implantation depth might directly affect these structures, predisposing patients to new-onset bundle branch block or high-grade atrioventricular block (8). Comprehending this anatomical relationship is essential for analyzing postprocedural ECG changes and for predicting the likelihood of permanent pacemaker implantation.

The impact of preprocedural and post-TAVI electrical abnormalities on both short- and long-term prognosis remains unclear. Understanding the implications of ECG changes can optimize patient management and improve clinical outcomes. This systematic review aims to assess ECG changes before and after TAVI, evaluate their predictive role for PPM-I, and examine the potential role of systematic periprocedural ECG monitoring.

## 2 Methods

### 2.1 Search strategy

A systematic literature review was conducted in April 2024 using PubMed, Web of Science, and JSTOR databases to identify studies evaluating ECG changes after TAVI and their role in predicting PPM-I. The search strategy combined Medical Subject Headings (MeSH) and free-text keywords, including “transcatheter aortic valve implantation,” “TAVI,” “TAVR,”” electrocardiogram changes,” “conduction abnormalities,” “pacemaker implantation,” “fast-track technique,” and “randomized controlled trials.” In addition, to comprehensively expand our search, we reviewed the reference lists of all eligible articles. Boolean operators (AND, OR) were used to further refine the results and exclude irrelevant studies. The search strategy also included terms related to minimalist or fast-track TAVI protocols to ensure comprehensive identification of studies addressing postprocedural monitoring strategies in early-discharge settings. However, none of the included studies provided specific data on fast-track TAVI cohorts; therefore, this subgroup could not be analyzed separately.

### 2.2 Inclusion and exclusion criteria

We included only studies published between 2001 and 2024 that involved human subjects and reported original data on ECG changes. Eligible study designs comprised randomized controlled trials, cohort studies, case–control studies, and observational analyses. Conference abstracts, editorials, opinions, and letters were excluded.

### 2.3 PRISMA diagram

**Figure 1:**
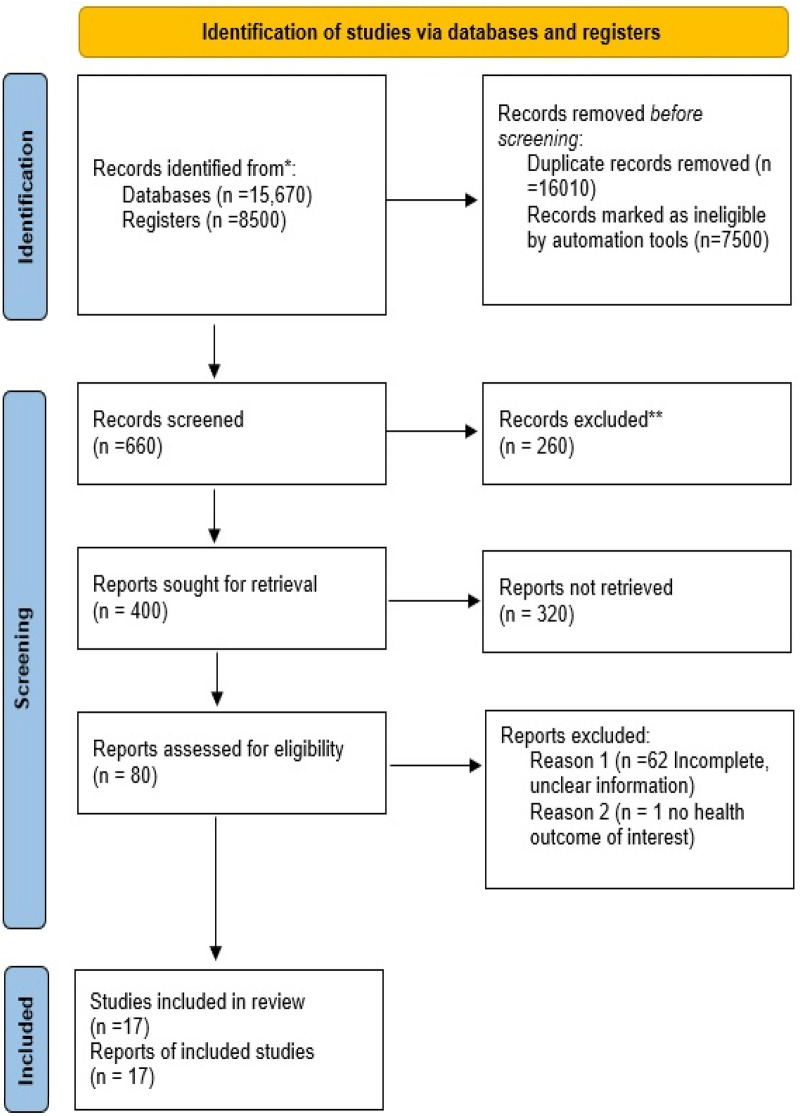
PRISMA diagram showing the study selection process.

The study selection process followed the Preferred Reporting Items for Systematic Reviews and Meta-Analyses (PRISMA) guidelines (31). Two independent reviewers screened all articles, and any discrepancies in study selection were resolved through consensus. A total of 24,170 records were identified, of which 16,010 were marked as duplicates and were removed from further processing. Of the remaining records, 7,500 were deemed unsuitable because they did not report original data, including review articles, systematic reviews, meta-analyses, conference abstracts, editorials, expert opinions, and letters to the editor, consistent with our predefined exclusion criteria. Of the remaining 400 records, an additional 320 did not meet the inclusion criteria and were excluded. Only records that addressed the “Impact of ECG changes following TAVI” were selected. A further 63 records were deemed incomplete or lacked clarity and were excluded. Ultimately, only 17 records met all inclusion criteria and were included in the study (Figure 1).

### 2.4 Risk of bias assessment

**Figure 2:**
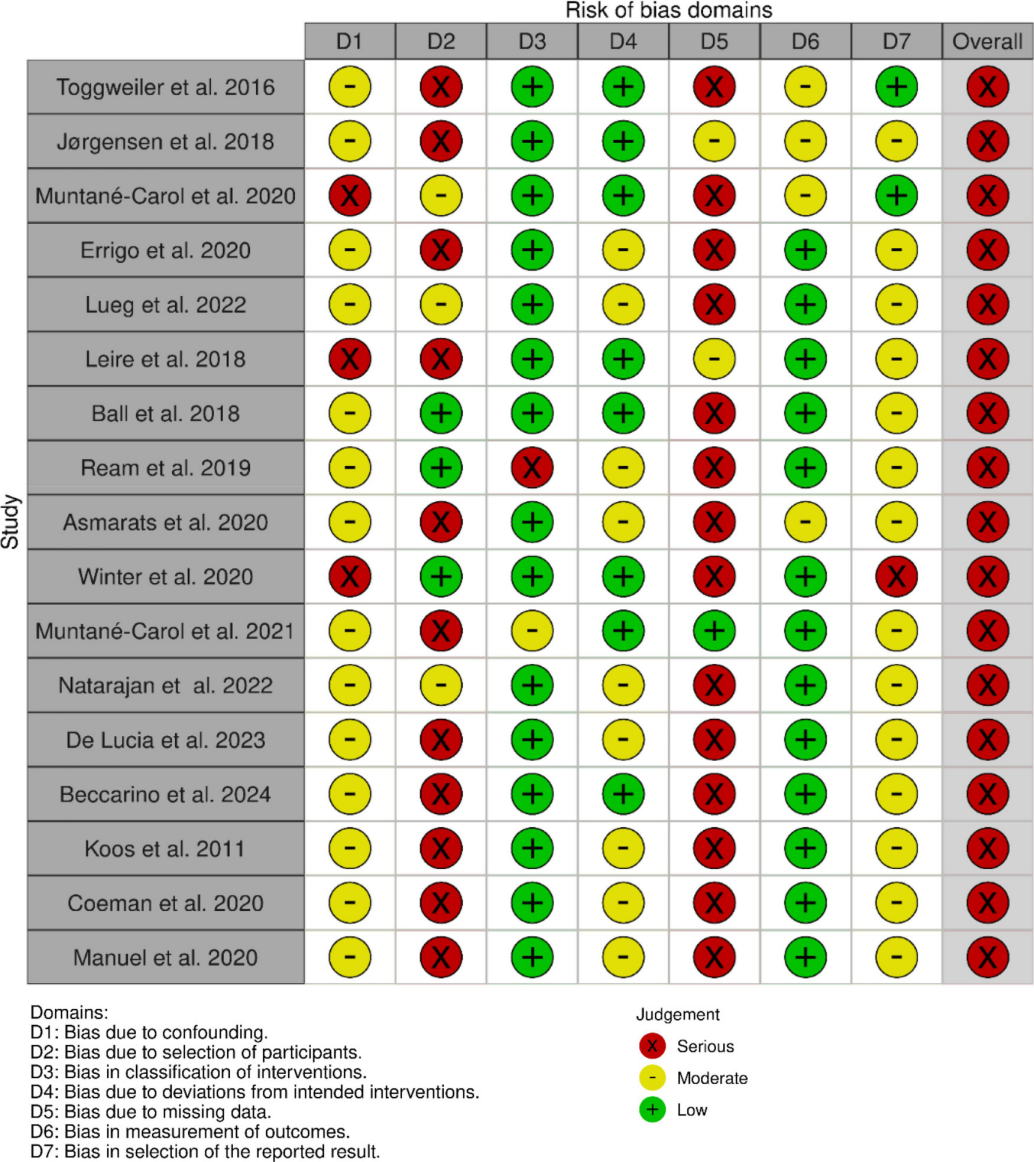
ROBINS-I tool used for bias risk assessment.

The risk of bias for each included study was assessed using the ROBINS-I tool (9), focusing on selection bias, misclassification of interventions, deviations from intended interventions, missing data, measurements of outcomes, and selection of the reported results (Figure 2).

Most studies included in this review were observational in design and therefore inherently at a moderate risk of confounding. Based on the ROBINS-I assessment, the primary concerns were related to selection bias, incomplete adjustment for procedural variables such as implantation depth or valve oversizing, and heterogeneity in monitoring duration and outcome definitions. Misclassification of interventions was generally low, as valve type and ECG criteria were consistently reported across studies. Outcome assessment bias was present in studies with limited follow-up duration or non-continuous monitoring strategies, which may have led to potential underestimation of late-onset conduction disturbances. Overall, the evidence base was characterized by a moderate risk of bias, which should be taken into account when interpreting the strength of associations between ECG changes and PPM implantation.

## 3 Results

The 17 studies that met the inclusion criteria provided insights into the occurrence, characteristics, and clinical implications of ECG changes before and after TAVI, particularly concerning conduction abnormalities and the subsequent need for PPM-I (Table 1).

**Table 1:** Study characteristics.

Study nr.	Authors	Methodology	Sample size	Objectives of the study	Success rate/measurement of treatment effect	Study implications
1	Toggweiler et al. (23)	Prospective	1,064	→ Identify indicators of delayed high-grade atrioventricular block (HAVB) and establish the necessity for and duration of telemetry monitoring	→ Pre-TAVI HAVB incidence of 8.7% → Delayed HAVB incidence of 6.7%, up to 8 days following TAVI → Patients with no conduction defects (CDs) immediately after TAVI showed no signs of delayed HAVB	→ Patients without immediate post-TAVI conduction abnormalities did not develop delayed AVB and may not require continuous telemetry monitoring
2	Ball et al. (15)	Retrospective	209	→ Determine which ECG characteristics are significant predictors for long-term pacing in patients	→ ECG parameters were comparable across individuals who underwent PPM-I within a week after the procedure (21.1%) compared to those who had none (78.9%)	→ Post-TAVI conduction delays predict the need for PPM-I
3	Jørgensen et al. (16)	Single-center	467	→ Determine early post-TAVI ECG indicators of delayed high-degree conduction defect (HD-CD) within 30 days after procedure	→ For patients in sinus rhythm without RBBB, late HD-CD developed in 0 of 70 patients with PR interval <200 ms and QRS interval <120 ms and in 5 of 109 patients with PR interval <240 ms and QRS interval <150 ms → Late HD-CD developed in 14 of 101 patients with PR interval ≥240 ms or QRS interval ≥150 ms. →late HD-CD developed in three of 49 patients with atrial fibrillation and no RBBB with QRS interval <140 and ≥140 ms, respectively	→ Immediate post-TAVI ECG findings are valuable for the early and safe removal of the temporary pacemaker in patients without RBBB who are in the sinus rhythm (PR interval <240 ms and QRS interval <150 ms) or atrial fibrillation (QRS interval <140 ms)
4	Leire et al. (13)	Single-center, prospective	76	→ Characterize ECG variations and conduction anomalies in patients undergoing TAVI	→ Post-TAVI temporary changes: prolonged PR interval, QRS complex widening, longer QTc, left QRS axis deviation, and abnormal T waves → Complete heart block (CHB) incidence of 2.9%, new-onset LBBB incidence of 39%	→ TAVI was related to a variety of transient post-TAVI conduction abnormalities → Post-TAVI, persistent LBBB was common
5	Muntané-Carol et al. (27)	Longitudinal, observational, single-center	397	→ Long-term assessment of outcomes in patients with no conduction abnormalities (CAs) on post-TAVI ECG.	→ PPM-I rate was 3.5% (1.1%/year) in the non-ECG-CA group vs. 15.7% (5.5%/year) in the ECG-CA group (*p* < 0.001) → Pre-existing CAs independently predicted a higher risk of PPM-I (HR 4.67 (95% CI 2.15-10.16), *p* < 0.001)	→ Most patients with no prior CAs had no notable post-TAVI ECG abnormalities → Pre-existing ECG CAs were associated with a high risk of heart failure hospitalization and PPM-I
6	Errigo et al. (28)	Single-center	431	→ Determine clinical, ECG, and procedural determinants of PPM-I following TAVI	→ 18% of patients needed PPM-I, either early (11%) or late-beyond the third day (7%). → Preoperative RBBB increased the risk of PPM-I by more than fivefold; using a self-expandable prosthesis tripled the risk → Syncope was significantly associated with an increased risk of late PPM-I	→ Pre-existing RBBB, self-expandable prostheses, and syncope may identify individuals at high risk
7	Lueg et al. (21)	Retrospective	850	→ Investigate ECG changes following TAVI with modern valves and identify clinical indicators to consider PPM-I according to ESC guidelines	→ Post-TAVI, 9.1% of patients developed new-onset LBBB with QRS >150 ms, and 3.1% exhibited new PR prolongation >240 ms → Calcification of the aortic annulus was associated with PR prolongation → New-onset LBBB and the need for PPM-I were associated with self-expandable valves	→ Per ESC guidelines, 25.1% of patients after TAVR would have required further evaluation → Self-expandable valves were associated with new-onset LBBB and PPM-I → Coronary heart disease and peripheral arterial disease correlated with QRS complex widening after TAVI → Risk factors for PPPM-I included pre-existing RBBB, first-degree AV block, prosthesis size, and implantation depth → New-onset LBBB after TAVI indicated a higher long-term risk for PPM-I
8	Ream et al. (10)	Single-center	150	→ Evaluate the effectiveness of ambulatory event monitoring (AEM) in detecting post-TAVI delayed high-grade atrioventricular block (DH-AVB) and related risk variables	→ DH-AVB was detected in 10% of patients, on average, at 6 days after TAVI → Patients with RBBB had a higher risk of developing DH-AVB, with a specificity and sensitivity of 27% and 94%, respectively	→ AEM is valuable in the early detection and management of 10% of post-TAVI outpatients who exhibited DH-AVB
9	Asmarats et al. (11)	Prospective, single-center	90	→Establish the prevalence and nature of unreported pre-TAVI arrhythmic events (AEs) via continuous electrocardiographic monitoring (CEM) → Assess the nature and effect of medical interventions resulting from the discovery of AEs	→ Severe bradyarrhythmias were noted in 36.4% of all patients; 18.2% required therapy changes →CEM identified AEs in 51 patients (48.1%) → Chronic renal failure, valve calcification, and left ventricular dysfunction were associated with a higher incidence of AEs before TAVI	→ Continuous pre-TAVI ECG monitoring significantly enhanced the detection of unknown AEs and led to therapeutic changes in patients before TAVI → The burden of AEs correlated with pre-existing CDs and chronic renal failure
10	Winter et al. (29)	Retrospective	62	→ Evaluate the role of remote ambulatory cardiac monitoring (rACM) in detecting pre- and post-TAVI HAVB	→ Using rACM, nearly 50% of PMM-I in TAVI patients were prospectively identified	→ Pre- and post-TAVI rACM identified nearly 50% of the patients who require PMM-I
11	Muntané-Carol et al. (12)	Prospective, multicenter	459	→ Assessment of the impact of delayed complete heart block (CHB) or high-grade atrioventricular block (HAVB) after TAVI using ambulatory ECG (AECG) monitoring	→ 4.6% of patients had delayed HAVB or CHB, out of which 81% required PPM-I.	→While HAVB or CHB were infrequent in patients lacking post-TAVI ECG abnormalities, underlying RBBB and sudden development of CDs identified those at higher risk
12	Natarajan, et al. (30)	Prospective cohort	192	→ Role of pre- and post-TAVI rACM in detecting conduction abnormalities → Potentially decreasing the unexpected need for predischarge PPM-I	→ ECG monitoring identified significant bradyarrhythmia in 7.3% of patients pre-TAVI and 9.2% post-TAVI. → New-onset AF was detected in 3.6% pre-TAVI and 3.9% post-TAVI.	→ rACM protocol with 2 weeks pre- and post-TAVR often resulted in the indication for PPMI, with significant patient adherence
13	De Lucia et al. (14)	Single-center, prospective, and non-controlled	163	→ Incidence at 30 days after TAVI delayed CDs through remote ECG monitoring	→ Delayed conduction disturbances (CDs), as indicators for PPM-I, were reported at a median of 6 days in 8% of patients, with 95% patient adherence to monitoring	→ 30-spot ambulatory ECG (KardiaMobile-6L) device is effective and safer in monitoring delayed CDs after TAVI
14	Beccarino et al. (24)	Multicenter, prospective	693	→ Assess the value of a standardized, systematic method using regular mobile cardiac telemetry (MCT) to promote secure and faster discharge by detecting conduction abnormalities that indicate a need for PPM-I	→ 3.0% of patients discharged after TAVI required PPM-I within 30 days → MCT monitoring identified patients with PPM-I indications (CHB, sick sinus syndrome, sinus bradycardia, pauses)	→ Consistent, systematic MCT after TAVI allows rapid identification of patients requiring PPM-I
15	Koos et al. (25)	Observational	80	→ Evaluate the role of ECG and imaging parameters in identifying patients requiring PPM-I after TAVI	→ Post-TAVI, 25% of patients developed new-onset LBBB → In 21% of patients (all CoreValve patients), PPM-I was required for CHB or complete RBBB or LBBB with AV delay → 67% of patients with preprocedural RBBB had an indication for PMM-I → Prosthesis design (*r* = 0.30, *p* = 0.01) and preprocedural RBBB (*r* = 0.4, *p* = 0.02) were strongly associated with PPM-I	→ The CoreValve ReValving system was associated with the greatest incidence of new-onset LBBB and PPM-I → Patients with preprocedural RBBB are at risk for PPM-I after TAVI
16	Coeman et al. (22)	Single-center	133	→ Determine the association between new-onset ECG changes and duration patterns based on valve design in balloon-expandable valve (BEV) and self-expandable valve (SEV) TAVI	→ PR interval of SEV recipients had a significant prolongation 48 h after TAVI: 33.7 ± 22.0 ms (*p* < 0.001)	→ Valve design is associated with the new-onset and variable timing of post-TAVI conduction abnormalities → SEV recipients had a higher rate of PPM-I beyond 24 h compared to BEV recipients
17	Manuel et al. (20)	Retrospective	182	→ Analyzed the incidence, type, and determinant of post-TAVI ECG abnormalities based on the prosthesis model	→ Self-expanding prostheses (SEP) were used in 54% of cases. → While 80% of the patients were initially in sinus rhythm, following TAVI, 21% developed new-onset AF →At discharge, the PR interval and QRS duration increased significantly, and 25% of patients exhibited new-onset LBBB → The depth of valve insertion was associated with new-onset LBBB at discharge	→Post-TAVI, PR prolongation, QRS widening, and new-onset LBBB were associated with SEP implantation → These abnormalities appeared to improve after 6 months →Deeper valve implantation predicted worse outcomes

After TAVI, the most frequently reported ECG abnormalities were new-onset left bundle branch block (LBBB), PR interval prolongation, and QRS complex widening, as summarized in Table 2. The incidence of new-onset LBBB varied across studies, ranging from 25% to 39%. Although some cases of new-onset LBBB resolved spontaneously before discharge, a significant proportion of patients with persistent LBBB were found to have an increased risk of requiring PPM-I.

**Table 2:** Summary of the results.

Study nr.	Study	ECG findings	Follow-up duration	Valve type	PPM-I rate
1	Toggweiler et al. (23)	Pre-TAVI HAVB 8.7%, delayed HAVB 6.7%, 8 days after TAVI	8 days	Mixed	21%
2	Ball et al. (15)	PR and QRS increased by >18.9% after TAVI	Post-TAVI monitoring	Mixed	21.1%
3	Jorgensen et al. (16)	PR >240 ms, QRS >150 ms = highest risk of late conduction defects	30 days	Mixed	19.1%
4	Leire et al. (13)	39% of patients developed new-onset LBBB	Short-term (until discharge)	Mixed	2.86%
5	Muntané-Carol et al. (27)	CHB/HAVB	Long-term (yearly follow-up)	Mixed	15.7% in the high-risk group 3.5% in the non-high-risk group
6	Errigo et al. (28)	Preoperative RBBB increased the risk of PPM by fivefold	Postprocedural monitoring	SEV	18%
7	Lueg et al. (21)	9.1% of patients developed new-onset LBBB, QRS >150 ms; PR >240 ms in 3.1% of patients	Post-TAVI monitoring	Mixed	3.3%
8	Ream et al. (10)	10% of patients developed delayed HAVB (>48 h after TAVI)	6 days (on average)	Mixed	12% in 2 days post-TAVI (20% in total)
9	Asmarats et al. (11)	Pre-existing RBBB significantly increased the risk of HAVB	Postprocedural monitoring	Mixed	18.9%
10	Winter et al. (29)	Remote ambulatory ECG detected HAVB in >50% of TAVI patients	Remote ECG monitoring	Mixed	12.1% pre-TAVI 12.1% post-TAVI
11	Muntané-Carol et al. (12)	Delayed HAVB/CHB in 4.6% of patients	Post-TAVI monitoring	Mixed	81%
12	Natarajan et al. (30)	RBBB with either a prolonged PR interval or a fascicular block	Pre- and post-TAVI monitoring	Mixed	3.6% pre-TAVI 4.6% post-TAVI based on rACM
13	De Lucia et al. (14)	PR prolongation	30 days	Mixed	8%
14	Beccarino et al. (24)	CHB, AV block Mobitz type II, bradycardia	30-day MCT monitoring	Mixed	16%
15	Koos et al. (25)	Post-TAVI, 25% of patients exhibited new-onset LBBB and RBBB emerged as a strong predictor	Post-TAVI monitoring	SEVs	21%
16	Coeman et al. (22)	SEV patients had delayed PR prolongation	48 h post-TAVI	SEVs vs. BEVs	15.3% (higher in SEVs)
17	Manuel et al. (20)	25% of patients exhibited new-onset LBBB, PR and QRS prolongation, and persistent QRS complex widening	6 months	SEVs	18%

PR interval prolongation, particularly when exceeding 240 ms, was another commonly reported finding. In such patients, significant PR prolongation often exhibited a higher probability of developing high-grade atrioventricular block (HAVB), often necessitating PPM-I. Similarly, QRS complex widening, particularly when exceeding 150 ms, was a strong predictor of delayed conduction abnormalities and was associated with the need for PPM-I. While PR interval prolongation showed modest improvement over long-term follow-up, QRS complex widening persisted at 6 months, confirming its importance as a marker of long-term conduction abnormality. Interestingly, Ball et al. reported that a ≥18.9% increase in the combined PR and QRS duration after TAVI was associated with a substantially increased risk of pacemaker implantation, with a sensitivity of 63% and specificity of 73%. Our post-TAVI ECG findings emphasize the need for postprocedural monitoring in such selected patients to assess the need for PPM-I.

Several studies also reported other conduction abnormalities after TAVI, although these were described less consistently than LBBB. Complete heart block was generally uncommon, with rates usually ranging between 3% and 8%. High-grade AV block showed a similar pattern, occurring in approximately 4%–10% of patients, depending on valve type and the duration of rhythm monitoring. New-onset right bundle branch block (RBBB) was mentioned in only a few reports and tended to be infrequent, approximately 3%–7%. Permanent pacemaker implantation rates varied widely across the included studies, ranging from 2.9%–3.5% in low-risk cohorts without baseline or postprocedural conduction abnormalities to 18%–21% in higher-risk populations, particularly among patients with pre-existing right bundle branch block or those treated with self-expanding valves. Taken together, these findings reflect the wide variation in conduction outcomes reported in the literature and underscore the need for individualized postprocedural monitoring strategies. Before TAVI, patients with pre-existing RBBB were consistently identified as being at higher risk of developing high-grade AV block after the procedure. Several studies reported that individuals with baseline RBBB had a substantially increased likelihood of requiring permanent pacemaker implantation (10, 11). This heightened vulnerability appears to be specific to RBBB or bifascicular block, as these patterns involve conduction pathways located closest to the region of valve deployment. Consequently, patients with RBBB generally warrant closer rhythm surveillance after TAVI. In contrast, other baseline conduction abnormalities—such as mild first-degree AV block or non-specific QRS prolongation—have not shown the same predictive strength and may not routinely require extended monitoring. Consistent with these findings, patients without baseline RBBB and without new postprocedural ECG changes have a much lower risk of late conduction complications (12).

The type of transcatheter valve was associated with observed postprocedural conduction abnormalities. Self-expanding valves (SEVs) were consistently associated with a higher incidence of conduction disturbances after TAVI compared with balloon-expandable valves (BEVs). Mechanistically, this phenomenon is likely linked to the prolonged pressure exerted by SEVs on the conduction system adjacent to the aortic valve annulus, reflecting the differences in the mechanical properties of SEVs vs. BEVs (10, 13). Patients treated with SEVs experienced delayed PR prolongation beyond 48 h following TAVI, while those receiving BEVs generally exhibited only minor changes in their PR interval. Patients who received SEVs experienced a significantly higher incidence of delayed PPM-I (>24 h post-TAVI) compared with BEV recipients. Our findings support extending the duration of ECG monitoring in patients treated with SEVs beyond the usual 48 h to identify delayed-onset conduction abnormalities that may lead to PPM-I. Most studies included in our review focused on earlier- or midgeneration TAVI devices, which are known to have higher rates of conduction disturbances. Emerging data suggest that newer valve platforms may reduce this risk; however, current evidence remains limited. In one recent comparison of older and newer transcatheter heart valves (THVs), the newer devices showed fewer new conduction abnormalities, but the overall 30-day pacemaker rates were not significantly different between generations. Among newer-generation devices, the Sapien 3 Ultra demonstrated markedly lower rates of both new-onset conduction disturbances (4.8% with Sapien 3 Ultra vs. 16.4% with CoreValve EP) and permanent pacemaker implantation (9.2% Sapien 3 Ultra vs. 24.9% CoreValve EP) (32). These observations suggest that specific design changes—such as modifications in frame geometry, radial force, or deployment control—may reduce mechanical stress on the conduction system. Even so, conduction outcomes remain strongly influenced by patient anatomy, implantation depth, and baseline conduction status. As more data become available, especially from head-to-head comparisons of specific valve platforms, we may gain a clearer understanding of how device iterations influence the need for postprocedural monitoring.

Remote ECG monitoring has emerged as a valuable tool for detecting delayed conduction abnormalities that might otherwise be missed by standard in-hospital telemetry (14). Extended ECG surveillance has shown that patients with persistent LBBB or progressive PR prolongation are at a significantly higher risk for late-onset HAVB and subsequent PPM-I. De Lucia et al. reported that 8% of patients undergoing non-continuous mobile ECG monitoring developed delayed CAs requiring PPM-I and found that PR interval prolongation at discharge was the strongest predictor. These findings underscore the need for prolonged ECG monitoring in selected high-risk individuals to ensure early detection and appropriate intervention.

## 4 Discussion

This systematic review highlights the critical role of post-TAVI ECG monitoring in the detection and management of commonly encountered CAs that may lead to PPM-I. The findings emphasize the necessity for standardized and prolonged monitoring protocols, particularly for patients at higher risk based on pre- and postprocedural ECG changes, as summarized in Table 3.

**Table 3:** High-risk patients for post-TAVI conduction abnormalities post-TAVI.

Risk category	Identified criteria	Increased risk for	Reference
Pre-TAVI ECG markers	RBBB	→ HAVB, delayed conduction defects	Muntané-Carol et al. (12), Coeman et al. (22)
	PR interval >200 ms (first-degree AV block)	→ Late-onset HAVB, PPM requirement	Ball et al. (15), Jørgensen et al. (16)
	QRS >120 ms (wide QRS complex)	→ Increased PPM-I risk, delayed conduction disturbances	Coeman et al. (22), Natarajan et al. (30)
	Bifascicular block (RBBB + LAFB or LPFB)	→ Progression to CHB, high PPM rate	Muntané-Carol et al. (12)
	Severe left ventricular outflow tract (LVOT) calcification	→ Mechanical injury to the conduction system, HAVB development	Coeman et al. (22)
	SEV implantation planned	→ Higher mechanical pressure on the conduction system leads to increased PPM rates	Muntané-Carol et al. (12)
Post-TAVI Early ECG Changes	New-onset LBBB	→ Persistent LBBB increases the risk of late-onset HAVB and PPM	Leire et al. (13)
	PR interval prolongation >240 ms	→ Late-onset HAVB and PPM requirement	Ball et al. (15)
	QRS >150 ms or a ≥18.9% increase in PR + QRS duration	→ Strongest predictor for PPM-I	Ball et al. (15)
	New HAVB (second or third degree)	→ Urgent need for PPM	Muntané-Carol et al. (12)
Delayed risk (post-TAVI >48 h–30 days)	Persistent new-onset LBBB (>48 h post-TAVI)	→ Late HAVB, increased mortality	Coeman et al. (22)
	Intermittent advanced AV block on ambulatory ECG monitoring	→ High risk of sudden cardiac arrest	De Lucia et al. (14)
	Prolonged PR interval + QRS widening beyond 72 h	→ Likely progression to HAVB	Natarajan et al. (30)
High-risk patient groups for extended ECG monitoring	Patients with pre-existing RBBB + PR prolongation	→Strongest predictor of late PPM requirement	Muntané-Carol et al. (12)
	Patients receiving SEV	→ Higher risk of conduction block requiring PPM	Muntané-Carol et al. (12)
	Patients with persistent conduction disturbances >48 h post-TAVI	→ High risk of late HAVB and PPM requirement	De Lucia et al. (14)

### 4.1 Pre-TAVI ECG: identifying high-risk patients

Pre-existing RBBB emerged as the strongest predictor of post-TAVI CAs and was consistently associated with an increased risk of high-degree AV block and the need for PPM-I (11). Furthermore, first-degree AV block (PR > 200 ms) and QRS complex widening (>120 ms) indicate conduction system impairment and vulnerability, particularly when present in combination with bifascicular block (15). A pre-existing left anterior fascicular block (LAFB) before TAVI also increases the likelihood of delayed CAs, serving as a precursor to late-onset heart block (16). Patients exhibiting these abnormalities are at an elevated risk and warrant more rigorous postprocedural ECG monitoring (15, 16). In addition to ECG-based predictors, prior studies have shown that anatomical and procedural factors—such as a short membranous septum, deeper valve implantation relative to the membranous septum, annular calcification, valve oversizing, and larger prosthesis size, particularly with self-expanding valves—are associated with a higher risk of conduction disturbances and permanent pacemaker implantation after TAVI (13, 17–19). These variables were not included in the present systematic analysis because they were not consistently reported or assessed across the studies we identified, most of which focused primarily on electrocardiographic findings.

### 4.2 Post-TAVI ECG: timing, duration, and predictive value of changes

The early post-TAVI period represents a critical window for detecting ECG CAs. New-onset LBBB is the most common finding and exhibits variable persistence related to valve type (20). Lueg et al. reported that persistent new-onset LBBB post-TAVI is associated with a higher probability of delayed HAVB and PPM-I (21). Conversely, new-onset LBBB may resolve spontaneously, highlighting the need for careful differentiation between transient and progressive conduction abnormalities (14). In addition, the combination of PR interval prolongation with QRS complex widening has been identified as a predictor of post-TAVI CAs. Coeman et al. demonstrated that an increase of 18.9% or more in the combined PR and QRS duration after TAVI significantly increases the likelihood of PPM-I (22). These findings underscore the importance of systematic remote or telemetry ECG evaluation after TAVI, particularly in patients who develop new-onset LBBB or exhibit a combination of PR prolongation and QRS complex widening (11).

### 4.3 Optimizing ECG monitoring strategies

The choice between scheduled ECG monitoring during outpatient clinic visits and continuous telemetry ECG monitoring remains insufficiently guided by the existing literature. The determination of the optimal duration and method of post-TAVI ECG monitoring remains a subject of ongoing research and must be tailored to the risk profile of each patient. While patients with normal ECG findings or transient CAs that resolve within 48 h may require only routine follow-up, those with persistent conduction abnormalities necessitate extended monitoring. Current recommendations suggest monitoring for at least 5–7 days in patients with persistent LBBB, prolonged PR intervals (>240 ms), or QRS complex widening (>150 ms) (23). However, the optimal monitoring duration is not standardized and should be individualized. Monitoring can usually be shortened once conduction intervals stabilize or begin to improve, and no episodes of higher-grade AV block are observed. Remote ECG monitoring and mobile cardiac telemetry have emerged as valuable tools for detecting delayed conduction abnormalities beyond the initial hospital stay and thus may improve outcomes (24).

Selecting between continuous telemetry and periodic ECG monitoring remains a key component of post-TAVI patient care. Continuous telemetry provides real-time detection of conduction abnormalities but requires significant resources. Research indicates that periodic, scheduled ECG evaluations at discharge, as well as at 1 week and 1 month after TAVI, may help identify patients at risk while minimizing hospital length of stay and overall costs (25). Ambulatory ECG monitoring is most beneficial for patients at higher risk of delayed conduction disturbances after TAVI, particularly those with pre-existing RBBB or bifascicular block, new-onset or persistent LBBB, marked PR prolongation (>240 ms), significant QRS complex widening (>150 ms), or interval progression during the first 24–48 h. Patients without these features generally do not require extended ECG monitoring.

**Figure 3:**
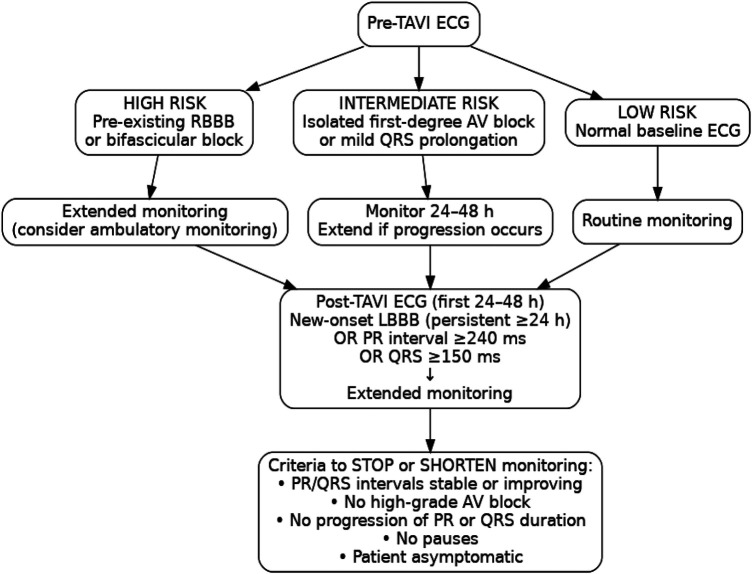
Proposed ECG-based monitoring algorithm before and after TAVI.

The role of electrophysiology study (EPS) is more limited. Although EPS can help characterize AV conduction in selected borderline cases, its ability to predict delayed high-grade AV block is modest, and current thresholds are not well validated (26). Ambulatory ECG monitoring is more effective in detecting late spontaneous AV block, which represents the predominant mechanism of delayed events. For this reason, ambulatory monitoring is preferred for most high-risk patients, with EPS reserved for cases in which diagnostic uncertainty persists despite serial ECG assessments. While fast-track or minimalist TAVI pathways have gained increasing adoption, particularly in low-risk patients, none of the included studies reported conduction outcomes specifically within this subgroup. Given that early discharge strategies rely heavily on accurate early risk stratification, future research should evaluate whether patients undergoing fast-track TAVI require distinct ECG monitoring algorithms, especially those with pre-existing RBBB, new-onset LBBB, or postprocedural PR/QRS prolongation. A flowchart has been constructed to synthesize the findings of this review into a practical monitoring algorithm (Figure 3).

### 4.4 Clinical implications and future perspectives

The findings of this systematic review highlight the need for a structured and individualized approach to post-TAVI ECG monitoring. By integrating pre-TAVI ECG findings with postprocedural ECG abnormalities, as well as anatomical and procedural factors, clinicians can tailor monitoring strategies according to individual patient risk profiles and distinguish between patients who need intensive monitoring and those who require limited follow-up after discharge. Such individualized strategies would result in optimized costs and a reduction of unnecessary PPM-I in patients with only transient CAs. The integration of wearable ECG monitoring technologies and artificial intelligence-based ECG analysis may further refine risk stratification and enable real-time assessment of CAs. Ongoing trials evaluating the impact of prolonged ECG monitoring on clinical outcomes are likely to help identify optimal post-TAVI monitoring strategies.

### 4.5 Limitations

We included mostly observational, retrospective, single-center studies, which limits the ability to confer direct causality, although a strong association between CAs and PPM-I was noted. Given the relatively small cohort size and the potential for selection bias, these findings cannot be generalized to a broader patient demographic and are applicable only to TAVI patients. Methodological inconsistencies, including variations in follow-up duration, monitoring techniques, and definitions of CAs, further limit applicability, even within the TAVI population. Some studies focused primarily on short-term ECG abnormalities, particularly immediate post-TAVI conduction abnormalities, and disregarded their long-term impact. These inconsistencies hinder data aggregation and further statistical analyses, which may have resulted in the development of either a clinical risk score tool or a standardized approach to choosing the type and duration of post-TAVI ECG monitoring. While differences related to valve design (BEVs vs. SEVs) were observed, their significance and clinical relevance remain difficult to interpret in the absence of direct comparative research. Finally, identified predictors of PPM-I, such as RBBB and valve implantation depth, were not universally validated across all investigations, potentially leading to variations in data implementation.

## 5 Conclusions

An individualized approach that integrates pre- and post-TAVI ECG changes can identify patients needing PPM-I. Our review underscores the need for systematic, structured, and patient-specific ECG monitoring protocols to determine individual risk. Such ECG protocols can only be the product of prospective, multicenter, large-cohort studies and may result in a change in our practice guidelines.

## Data Availability

The original contributions presented in the study are included in the article/Supplementary Material, further inquiries can be directed to the corresponding author.

## References

[1] Otto CM Prendergast B. Aortic-valve stenosis–from patients at risk to severe valve obstruction. N Engl J Med. (2014) 371(8):744–56. 10.1056/NEJMra1313875 25140960

[2] Campante Teles R Gama Ribeiro V Patrício L Neves JP Vouga L Fragata J Position statement on transcatheter aortic valve implantation in Portugal. Rev Port Cardiol. (2013) 32(10):801–5. 10.1016/j.repc.2013.02.009 23916790

[3] Vahanian A Beyersdorf F Praz F Milojevic M Baldus S Bauersachs J 2021 ESC/EACTS guidelines for the management of valvular heart disease. Eur Heart J. (2022) 43(7):561–632. 10.1093/eurheartj/ehab395 34453165

[4] Leon MB Smith CR Mack MJ Makkar RR Svensson LG Kodali SK Transcatheter or surgical aortic-valve replacement in intermediate-risk patients. N Engl J Med. (2016) 374(17):1609–20. 10.1056/NEJMoa1514616 27040324

[5] Reardon MJ Van Mieghem NM Popma JJ Kleiman NS Søndergaard L Mumtaz M Surgical or transcatheter aortic-valve replacement in intermediate-risk patients. N Engl J Med. (2017) 376(14):1321–31. 10.1056/NEJMoa1700456 28304219

[6] Auffret V Puri R Urena M Chamandi C Rodriguez-Gabella T Philippon F Conduction disturbances after transcatheter aortic valve replacement: current status and future perspectives. Circulation. (2017) 136(11):1049–69. 10.1161/CIRCULATIONAHA.117.028352 28893961

[7] Urena M Webb JG Cheema A Serra V Toggweiler S Barbanti M Impact of new-onset persistent left bundle branch block on late clinical outcomes in patients undergoing transcatheter aortic valve implantation with a balloon-expandable valve. JACC Cardiovasc Interv. (2014) 7(2):128–36. 10.1016/j.jcin.2013.08.015 24440024

[8] Poels TT Stassen R Kats S Veenstra L van Ommen V Kietselaer B Effective distance between aortic valve and conduction system is an independent predictor of persistent left bundle branch block during transcatheter aortic valve implantation. Medicina (Kaunas). (2021) 57(5):476. 10.3390/medicina57050476 34064932 PMC8150689

[9] McGuinness LA Higgins JPT. Risk-of-bias VISualization (robvis): an R package and shiny web app for visualizing risk-of-bias assessments. Res Synth Methods. (2021) 12(1):55–61. 10.1002/jrsm.1411 32336025

[10] Ream K Sandhu A Valle J Weber R Kaizer A Wiktor DM Ambulatory rhythm monitoring to detect late high-grade atrioventricular block following transcatheter aortic valve replacement. J Am Coll Cardiol. (2019) 73(20):2538–47. 10.1016/j.jacc.2019.02.068 31118148

[11] Asmarats L Nault I Ferreira-Neto AN Muntané-Carol G Del Val D Junquera L Prolonged continuous electrocardiographic monitoring prior to transcatheter aortic valve replacement: the PARE study. JACC Cardiovasc Interv. (2020) 13(15):1763–73. 10.1016/j.jcin.2020.03.031 32682674

[12] Muntané-Carol G Okoh AK Chen C Nault I Kassotis J Mohammadi S Ambulatory electrocardiographic monitoring following minimalist transcatheter aortic valve replacement. JACC Cardiovasc Interv. (2021) 14(24):2711–22. 10.1016/j.jcin.2021.08.039 34949396

[13] Leire U Eulogio G Francisco José RR Francisco Javier PJ Juan MP Belen D-A Electrocardiographic changes and conduction disturbances after transfemoral aortic valve implantation with Edwards Sapien 3 prosthesis. J Electrocardiol. (2018) 51(3):416–21. 10.1016/j.jelectrocard.2018.02.009 29530523

[14] De Lucia R Giannini C Parollo M Barletta V Costa G Giannotti Santoro M Non-continuous mobile electrocardiogram monitoring for post-transcatheter aortic valve replacement delayed conduction disorders put to the test. Europace. (2023) 25(3):1116–25. 10.1093/europace/euac285 36691737 PMC10062351

[15] Ball TN Vasudevan A Mi Ko J Assar MD McCullough PA Stoler RC. Analysis of electrocardiographic intervals before and after transcatheter aortic valve implantation to predict the need for permanent pacing. Proc Bayl Univ Med Cent. (2018) 31(4):407–13. 10.1080/08998280.2018.1471884 30948968 PMC6413979

[16] Jørgensen TH De Backer O Gerds TA Bieliauskas G Svendsen JH Søndergaard L. Immediate post-procedural 12-lead electrocardiography as predictor of late conduction defects after transcatheter aortic valve replacement. JACC Cardiovasc Interv. (2018) 11(15):1509–18. 10.1016/j.jcin.2018.04.011 30093055

[17] Baraka M Kamal D Mostafa AE. Depth of implantation in relation to membranous septum as a predictor of conduction disturbances after transcatheter aortic valve implantation. Indian Pacing Electrophysiol J. (2024) 24:133–9. 10.1016/j.ipej.2024.03.003 38548225 PMC11143730

[18] Høydahl MP Kjønås D Rösner A Trones Antonsen B Forsdahl SH Predictors of permanent pacemaker implantation after transcatheter aortic valve implantation. Scand Cardiovasc J. (2025) 59:2481175. 10.1080/14017431.2025.2481175 40094972

[19] Chen Y-H Chang H-H Liao T-W Leu H-B Chen I-M Chen P-L Membranous septum length predicts conduction disturbances following transcatheter aortic valve replacement. J Thorac Cardiovasc Surg. (2022) 164:42–51.e2. 10.1016/j.jtcvs.2020.07.072 32891451

[20] Manuel AM Almeida J Guerreiro C Dias T Barbosa A Teixeira P The effects of transcatheter aortic valve implantation on cardiac electrical properties. Rev Port Cardiol. (2020) 39(8):431–40. 10.1016/j.repc.2020.02.011 32773138

[21] Lueg J Morell L Juri B Janiszewski A Hajduczenia M Hennig P Electrocardiographic changes after TAVR and their clinical impact according to new ESC pacing guidelines. Eur Heart J. (2022) 43(Supplement_2):ehac544.396. 10.1093/eurheartj/ehac544.396

[22] Coeman M Kayaert P Philipsen T Calle S Gheeraert P Gevaert S Different dynamics of new-onset electrocardiographic changes after balloon- and self-expandable transcatheter aortic valve replacement: implications for prolonged heart rhythm monitoring. J Electrocardiol. (2020) 59:68–73. 10.1016/j.jelectrocard.2020.01.005 32007908

[23] Toggweiler S Stortecky S Holy E Zuk K Cuculi F Nietlispach F The electrocardiogram after transcatheter aortic valve replacement determines the risk for post-procedural high-degree AV block and the need for telemetry monitoring. JACC Cardiovasc Interv. (2016) 9(12):1269–76. 10.1016/j.jcin.2016.03.024 27339844

[24] Beccarino N Epstein LM Khodak A Mihelis E Pagan E Kliger C The utility and impact of outpatient telemetry monitoring in post-transcatheter aortic valve replacement patients. Cardiovasc Revasc Med. (2024) 64:15–20. 10.1016/j.carrev.2024.02.012 38388248

[25] Koos R Mahnken AH Aktug O Dohmen G Autschbach R Marx N Electrocardiographic and imaging predictors for permanent pacemaker requirement after transcatheter aortic valve implantation. J Heart Valve Dis. (2011) 20(1):83–90. PMID: 21404902. 21404902

[26] Siontis KC Kara Balla A Cha Y-M Pilgrim T Sweda R Roten L Invasive electrophysiological testing to predict and guide permanent pacemaker implantation after transcatheter aortic valve implantation: a meta-analysis. Heart Rhythm O2. (2023) 4:24–33. 10.1016/j.hroo.2022.10.007 36713040 PMC9877393

[27] Muntané-Carol G Alméndarez M Junquera L Wintzer-Wehekind J Del Val D Faroux L Long-term electrocardiographic changes and clinical outcomes of transcatheter aortic valve implantation recipients without new postprocedural conduction disturbances. Am J Cardiol. (2020) 125(1):107–13. 10.1016/j.amjcard.2019.09.047 31732136

[28] Errigo D Golzio PG D’Ascenzo F Ragaglia E Bruno F Salizzoni S Electrocardiographic and clinical predictors for permanent pacemaker requirement after transcatheter aortic valve implantation: a 10-year single center experience. J Cardiovasc Surg (Torino). (2021) 62(2):169–74. 10.23736/S0021-9509.20.11342-9 32885926

[29] Winter JL Healey JS Sheth TN Velianou JL Schwalm J-D Smith A Remote ambulatory cardiac monitoring before and after transcatheter aortic valve replacement. CJC Open. (2020) 2(5):416–9. 10.1016/j.cjco.2020.04.006 32995727 PMC7499381

[30] Natarajan MK Sheth TN Wijeysundera HC Chavarria J Rodes-Cabau J Velianou JL Remote ECG monitoring to reduce complications following transcatheter aortic valve implantations: the redirect TAVI study. Europace. (2022) 24(9):1475–83. 10.1093/europace/euac042 35699482

[31] Page M McKenzie J Bossuyt P Boutron I Hoffmann T Mulrow CD The PRISMA 2020 statement: an updated guideline for reporting systematic reviews. Br Med J. (2021) 372:n71. 10.1136/bmj.n71 33782057 PMC8005924

[32] Salem M Laing P Kühling-thees I Kasper W Voran J Seoudy H Incidence of post-procedural conduction disturbances and rates of permanent pacemaker implantation in older and newer generations of transcatheter aortic heart valves. Med Sci (Basel). (2025) 13:296. 10.3390/medsci13040296 41440528 PMC12734965

